# DHA in Pregnant and Lactating Women from Coastland, Lakeland, and Inland Areas of China: Results of a DHA Evaluation in Women (DEW) Study

**DOI:** 10.3390/nu7105428

**Published:** 2015-10-21

**Authors:** You Li, Hong-tian Li, Leonardo Trasande, Hua Ge, Li-xia Yu, Gao-sheng Xu, Man-xi Bai, Jian-meng Liu

**Affiliations:** 1 Institute of Reproductive and Child Health/Ministry of Health Key Laboratory of Reproductive Health, Peking University Health Science Center, 38 Xueyuan Rd, Beijing 100191, China; liyou@pku.edu.cn (Y.L.); liht@bjmu.edu.cn (H.L.); 2 Department of Epidemiology and Biostatistics, School of Public Health, Peking University Health Science Center, 38 Xueyuan Rd, Beijing 100191, China; 3 Department of Pediatrics, NYU School of Medicine, 227 East 30th Street, Room 735, New York, NY 10016, USA; Leonardo.Trasande@nyumc.org; 4 Department of Obstetrics and Gynaecology, the First Affiliated Hospital of Baotou Medical School, 41 Linyin Rd, Baotou 014000, China; byyfygh2010@163.com; 5 Department of Obstetrics and Gynaecology, Weihai Maternal and Child Health Hospital, 51 Guangming Rd, Weihai 264200, China; whyulixia@126.com; 6 Department of Pediatrics, Yueyang Maternal and Child Health Hospital, 693 Baling Middle Rd, Yueyang 414000, China; xugaosheng0414@163.com; 7 Wyeth Nutrition Science Center, 582 Wuzhong Rd, Shanghai 201103, China; Manxi.Bai@wyethnutrition.com

**Keywords:** docosahexaenoic acid, pregnant women, lactating women, plasma, erythrocyte, correlation

## Abstract

Few studies have examined docosahexaenoic acid (DHA) in pregnant and lactating women in developing countries like China, where DHA-enriched supplements are increasingly popular. We aimed to assess the DHA status among Chinese pregnant and lactating women residing areas differing in the availability of aquatic products. In total, 1211 women in mid-pregnancy (17 ± 2 weeks), late pregnancy (39 ± 2 weeks), or lactation (42 ± 7 days) were enrolled from Weihai (coastland), Yueyang (lakeland), and Baotou (inland) city, with approximately 135 women in each participant group by region. DHA concentrations were measured using capillary gas chromatography, and are reported as weight percent of total fatty acids. Mean plasma DHA concentrations were higher in coastland (mid-pregnancy 3.19%, late pregnancy 2.54%, lactation 2.24%) and lakeland women (2.45%, 1.95%, 2.26%) than inland women (2.25%, 1.67%, 1.68%) (*p* values < 0.001). Similar differences were observed for erythrocyte DHA. We conclude that DHA concentrations of Chinese pregnant and lactating women are higher in coastland and lakeland regions than in inland areas. DHA status in the study population appears to be stronger than populations from other countries studied to date.

## 1. Introduction

Docosahexaenoic acid (DHA, 22:6n-3), as a fundamental constituent in cell membranes, is indispensable to the structure and function of the retina and central nervous system [1,2]. DHA is mainly contained in aquatic products, especially in seafood. Dietary DHA intake is a major source to meet human body requirements, since humans only synthesize a limited amount of DHA from α-linolenic acid [3]. It is widely acknowledged that pregnant and lactating women are more susceptible to DHA deficiency because they need to meet their own needs as well as those of the fetuses. Increased intake of DHA during pregnancy and lactation has been documented to benefit fetal and infant development [4,5,6,7].

The availability and consumption of aquatic products plays an important role in DHA status. In a study [8] conducted among women from four Tanzanian tribes differing in lifetime intakes of fish, Luxwolda *et al.* observed an obvious positive correlation between fish consumption and DHA levels. DHA status may also vary across ethnicities. In a study [9] comparing plasma DHA phospholipids between Dutch and ethnic minority pregnant women in Netherlands, van Eijsden *et al.* reported significant ethnic differences in maternal DHA status despite controlling for fish intake. Besides, in an earlier study [10] involving women from Ecuador and four European countries with different baseline phospholipid DHA status, Otto *et al.* observed consistent decreases in the DHA weight percentage of total fatty acids as women progress from early pregnancy to delivery.

In China, fish availability varies considerably in populations, and is greatest in coastland and lakeland regions in contrast to inland areas. In this study, we aimed to examine DHA status in a diverse population of Chinese pregnant and lactating women from coastland, lakeland, and inland areas.

## 2. Subjects and Methods

### 2.1. Settings and Subjects

The DHA Evaluation in Women (DEW) study was a cross-sectional survey conducted from May to July 2014 in three cities of China: Weihai (selected to represent the coastland population), Yueyang (selected to represent the lakeland population) and Baotou (selected to represent the inland population). Weihai is surrounded on three sides by the Huang Sea. Yueyang is near Dongting Lake, the second largest freshwater lake in China. Baotou is a typical inland city in the Mongolian Plateau. A total of 1211 apparently healthy women who were at mid-pregnancy (17 ± 2 gestational weeks), late pregnancy (39 ± 2 gestational weeks), or lactation (42 ± 7 days postpartum) were recruited approximately equally from the three regions, with on average 135 (127–138) women in each group per region. Eligible women were 18–35 years old, were local permanent residents, and had singleton pregnancies. An additional inclusion criterion for the lactating group was current breastfeeding. Women were excluded if they had been diagnosed with any cardiovascular, metabolic, and renal diseases, mental disorder, or aquatic food allergy; or had participated in other research projects in the past 30 days. Women with severe vomiting after 16 weeks of gestation were also excluded for the mid-pregnancy group. The research protocol was approved by the Institutional Review Boards/Human Subjects Committees at Peking University Health Science Center (IRB00001052-14012; date of approval: 22-04-2014), and all participating women signed informed consents.

### 2.2. Data and Sample Collection

Participants were enrolled from four local hospitals: one located in Weihai, one in Yueyang, and two in Baotou. Trained obstetricians or nurses from the hospitals completed enrolment and data collection. A structured questionnaire was used to collect maternal characteristics, including birthdate, ethnicity, height, pre-pregnancy weight, and educational attainment. For pregnant women, gestational age at enrolment was calculated according to the date of the last menstrual period. For lactating women, self-reported gestational age at delivery and parity were also collected.

Fasting venous blood (~5 mL) was collected from the antecubital vein into ethylenediaminetetraacetic acid (EDTA)-containing tubes. Samples were kept in the refrigerator at 5 °C for at least 30 min and then centrifuged at 3000× g for 10 min to separate plasma and erythrocytes. The erythrocytes were washed out with normal saline. Both plasma and erythrocyte samples were stored at −20 °C in the hospital for approximately 10 days, and then were transported on dry ice frozen at −80 °C to the central laboratory where samples were stored at a −80 °C freezer. Notably, the temporal storage of blood samples at −20 °C might have somewhat compromised DHA in erythrocyte [11,12].

To ensure data quality and to standardize data collection methodologies across sites, study staff attended training workshops and each site had a designated investigator who oversaw the standardized data collection procedures. In addition, senior investigators met weekly and provided additional oversight.

### 2.3. Sample Analysis

The extraction and derivatization of total lipids in plasma and erythrocyte samples were carried out using a modified method of Folch *et al.* [13]. The internal standard solution containing methyl undecanoate (C11:0) was added to the samples, and mixed with boron trifluoride and methanol. This mixture was heated at 115 °C for 20 min. After cooling to room temperature, the mixture was extracted with n-hexane. The n-hexane containing methyl esters of total lipids were analyzed by an Agilent 6890N gas chromatography (Agilent Technologies, Palo Alto, CA, USA) equipped with a flame ionization detector at 280 °C and a capillary column (CP-Sil 88, 50 m, 0.25 mm ID, 0.20 μm film thickness). The injector was set as a split mode at 250 °C, with the split ratio of 1:5. The oven temperature was programmed as follows: ramping from 120 °C to 166 °C at 2 °C/min, and holding at 166 °C for 10 min; then ramping to 200 °C at 2 °C/min and holding at 200 °C for 10 min. Individual fatty acids were identified against the reference standards. The data were collected and processed using Agilent OpenLAB software (Agilent Technologies, Santa Clara, CA, USA). Both absolute concentration (μg/mL) and the relative concentration (weight percent of total fatty acids, wt. %) of DHA were calculated.

### 2.4. Statistical Analysis

DHA concentrations are presented as means ± SDs. One-way analyses of variance were performed to compare overall differences in DHA concentrations among participant groups and regions. *T*-tests were used to examine the differences between women in inland and lakeland/coastland as well as between women in mid-pregnancy and late-pregnancy/lactation. Additionally, we explored whether DHA concentrations varied across subgroups based on maternal age (18.0–24.9, 25.0–29.9, and 30.0–34.9 years), pre-pregnancy BMI (<18.5, 18.5–23.9, and ≥24.0 kg/m^2^), and education attainment (middle school or less, high school, and college or above) by using multiple linear regression with adjustments for covariates including region and participant group.

To illustrate the relationship between plasma and erythrocyte DHA, we performed several sets of Pearson correlation analyses. We first estimated the overall correlation coefficient between plasma and erythrocyte relative DHA concentrations. Because the scatterplot indicated an obviously different correlation pattern between individuals with erythrocyte DHA concentrations ≥3% and those <3%, separate correlation analyses for the two subgroups were then performed. We also repeated the above-mentioned correlation analyses within the 9 subgroups defined by region and participant group.

Significance was set at *p* < 0.05. All statistical analyses were performed by using SPSS version 20.0 (Chicago, IL, USA).

## 3. Results

### 3.1. Maternal Characteristics

Table 1 shows maternal characteristics by region and participant group. Overall, 87.8% women had high school or above education. In coastland and lakeland populations, 98.0% were of Han ethnicity, whereas the percentage was somewhat lower (89.8%) in inland, where 7.1% were Mongolian. The mean age, height, and pre-pregnancy BMI were comparable for the three participant groups residing coastland and inland, whereas women from the lakeland were younger, shorter in stature, and had lower pre-pregnancy BMI (*p* values < 0.001). In the lactating group, primiparous women accounted for 87.5%, 81.5%, and 86.0% in coastland, lakeland, and inland, respectively, and corresponding mean gestational age at delivery in the three regions was 39.3, 39.3, and 39.1 weeks, respectively.

**Table 1:** Maternal characteristics by region and participant group.

	Coastland			Lakeland			Inland		
Characteristic	MP	LP	LA	MP	LP	LA	MP	LP	LA
Number of participants	136	127	136	133	134	135	138	136	136
GA (week)/PP (day) at enrolment									
Mean	16.9	37.5	42.7	17.0	38.0	41.7	16.7	38.6	42.1
SD	0.9	0.7	2.3	1.1	0.9	4.1	1.1	1.2	3.9
Age (year)									
Mean	27.9	28.4	28.3	26.5	27.1	27.1	27.9	28.5	28.1
SD	2.4	2.7	2.7	3.1	3.1	3.0	2.9	3.3	3.0
Ethnics (%)									
Han	97.8	99.2	97.1	98.5	97.8	97.8	83.3	94.1	91.9
Mongolian	0	0	0	0	0	0	12.3	4.4	4.4
Hui	0	0	0.7	0	0	0	0.7	0.7	2.2
Others	2.2	0.8	2.2	1.5	2.2	2.2	3.6	0.7	1.5
Education (%)									
College or above	67.7	66.2	65.4	47.4	61.3	62.2	79.0	78.4	73.5
High school	15.4	24.4	25.0	36.8	24.6	23.7	13.8	16.2	19.1
Middle school or less	16.9	9.4	9.6	15.8	14.1	14.1	7.2	15.4	7.4
Height (cm)									
Mean	163.6	163.7	163.2	159.3	159.8	159.8	164.1	162.8	163.1
SD	4.9	4.6	5.0	4.5	3.9	4.3	4.9	4.7	4.6
Pre-pregnancy BMI (kg/m^2^)									
Mean	21.1	21.3	21.9	20.2	19.8	20.1	21.3	21.6	21.2
SD	2.8	2.5	3.5	2.5	1.9	2.9	3.3	2.8	3.2

GA, gestational age; PP, postpartum; MP, mid-pregnancy; LP, late pregnancy; LA, lactation.

### 3.2. DHA Concentrations

Mean plasma and erythrocyte relative DHA concentrations of 9 region- and group-specific subgroups ranged 1.67%–3.19% and 5.06%–7.59%, respectively; corresponding absolute values ranged between 60.5 and 146.2 μg/mL and 89.3–127.7 μg/mL, respectively (Table 2).

**Table 2:** Docosahexaenoic acid (DHA) concentrations by region and participant group.

	Inland		Lakeland		Coastland		*P* ANOVA
	Mean	SD	Mean	SD	Mean	SD	
**wt. %**							
Plasma							
Mid-pregnancy	2.25	0.46	2.45 ^a^	0.44	3.19 ^a^	0.65	<0.001
Late pregnancy	1.67 ^b^	0.35	1.95 ^a,b^	0.45	2.54 ^a,b^	0.60	<0.001
Lactation	1.68 ^b^	0.48	2.26 ^a,b^	0.53	2.24 ^a,b^	0.70	<0.001
*P* ANOVA	<0.001		<0.001		<0.001		
Erythrocyte							
Mid-pregnancy	5.85	1.06	6.34 ^a^	0.80	7.59 ^a^	1.46	<0.001
Late pregnancy	5.06 ^b^	1.25	6.23 ^a^	1.09	7.09 ^a,b^	1.93	<0.001
Lactation	5.20 ^b^	1.15	6.20 ^a^	0.92	6.07 ^a,b^	1.59	<0.001
*P* ANOVA	<0.001		0.45		<0.001		
**μg/mL**							
Plasma							
Mid-pregnancy	93.4	25.2	86.2 ^a^	20.5	118.4 ^a^	30.8	<0.001
Late pregnancy	101.9 ^b^	33.1	110.1 ^a,b^	28.3	146.2 ^a,b^	45.1	<0.001
Lactation	60.5 ^b^	17.5	65.1 ^b^	20.9	75.7 ^a,b^	29.9	<0.001
*P* ANOVA	<0.001		<0.001		<0.001		
Erythrocyte							
Mid-pregnancy	108.0	22.1	103.2 ^a^	13.6	127.7 ^a^	27.2	<0.001
Late pregnancy	89.3 ^b^	25.2	106.6 ^a^	20.7	123.2 ^a^	37.5	<0.001
Lactation	91.4 ^b^	22.1	97.0 ^a,b^	19.7	99.3 ^a,b^	29.6	<0.05
*P* ANOVA	<0.001		<0.001		<0.001		

ANOVA, analyses of variance. ^a^: Mean values were significant different compared with women from inland within the same participant group (by *t*-test, *p* < 0.05); ^b^: mean values were significant different compared with women in mid-pregnancy within the same region (by *t*-test).

Plasma and erythrocyte DHA relative concentrations differed by region across participant groups (*p* values < 0.001). The concentrations were higher in coastland and lakeland women than in inland women. Similar regional differences in DHA absolute concentrations were observed (Table 2).

Plasma DHA relative concentrations differed significantly by participant groups across the regions (*p* values < 0.001); the concentrations were higher in mid-pregnancy than in late pregnancy and lactating women across regions (*p* values < 0.001). Similar patterns were also observed for erythrocyte DHA relative concentrations, although the difference was not significant among the three groups of lakeland women. In contrast, the plasma DHA absolute concentrations were highest in late-pregnancy women, followed by in mid-pregnancy, and lowest in lactating women. The erythrocyte DHA absolute concentrations were relatively higher in mid-pregnancy in inland, in mid- and late-pregnancy in lakeland and coastland women (Table 2).

In multiple linear regression analyses, maternal age and education were significantly associated with DHA concentrations; patterns for regional and inter-group DHA were similar to the aforementioned unadjusted analyses (Table 3).

### 3.3. Correlation between DHA in Plasma and Erythrocyte

**Figure 1:**
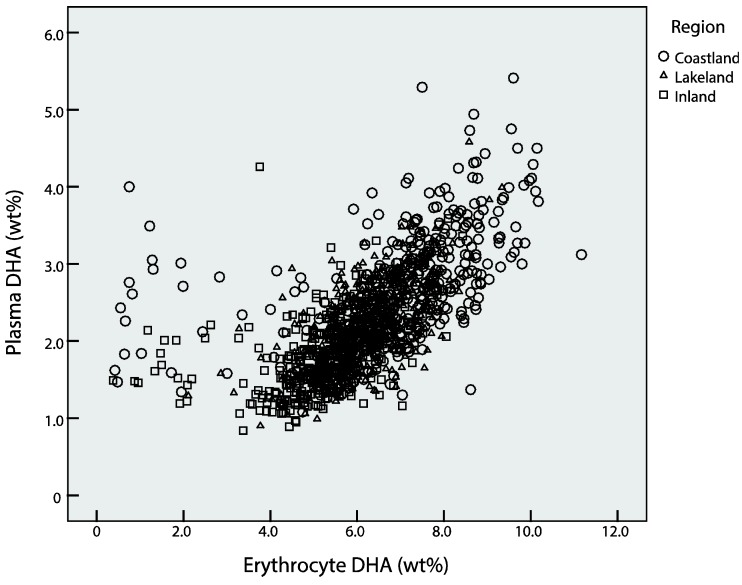
Correlation between plasma and erythrocyte DHA relative concentrations in 1211 participants.

The overall Pearson correlation coefficient between plasma and erythrocyte relative DHA concentrations was 0.625 (*p* < 0.001). The correlation profile was visually examined via a scatterplot and an obviously different correlation pattern was detected between women with erythrocyte DHA concentration ≥3% and those <3% (Figure 1). The significant positive correlation persisted only in women with erythrocyte DHA concentrations ≥3% (*n* = 1175; *r* = 0.73, *p* < 0.001), but not in those <3% (*n* = 36; *r* = −0.13, *p* = 0.46). After excluding women with erythrocyte DHA concentrations <3%, most of region- and group-specific correlation coefficients were substantially augmented by 18%–64% (Supplementary Table S1).

**Table 3:** Multiple linear regression of DHA concentrations on region, participant group and selected maternal characteristics.

Variables	Plasma DHA (wt. %)			Erythrocyte DHA (wt. %)			Plasma DHA (μg/mL)			Erythrocyte DHA (μg/mL)		
	Mean	β	*P*	Mean	β	*P*	Mean	β	*P*	Mean	β	*P*
Region												
Inland	1.87	0	Ref.	5.37	0	Ref.	85.3	0	Ref.	96.3	0	Ref.
Lakeland	2.22	0.36	<0.001	6.25	0.92	<0.001	87.0	3.6	0.091	102.2	6.7	<0.001
Coastland	2.66	0.80	<0.001	6.91	1.55	<0.001	112.7	28.4	<0.001	116.6	20.4	<0.001
Participant group												
Mid-pregnancy	2.63	0	Ref.	6.59	0	Ref.	99.4	0	Ref.	113.0	0	Ref.
Late pregnancy	2.04	−0.59	<0.001	6.10	−0.49	<0.001	118.8	19.4	<0.001	106.0	−7.0	<0.001
Lactation	2.06	−0.58	<0.001	5.82	−0.79	<0.001	67.1	−32.9	<0.001	95.9	−17.5	<0.001
Age(year)												
<25.0	2.15	0	Ref.	5.93	0	Ref.	88.2	0	Ref.	100.2	0	Ref.
25.0–29.9	2.27	0.02	0.623	6.24	0.17	0.129	94.7	1.7	0.499	106.1	2.8	0.191
≥30.0	2.25	0.12	0.029	6.15	0.25	0.050	100.6	7.9	0.006	105.3	4.0	0.108
Education												
Middle school or less	2.18	0	Ref.	5.93	0	Ref.	90.1	0	Ref.	100.2	0	Ref.
High school	2.24	0.07	0.187	6.20	0.29	0.033	94.2	7.2	0.016	105.0	5.6	0.034
College or above	2.26	0.12	0.014	6.21	0.36	0.003	96.0	8.5	0.002	105.8	6.4	0.006
Pre-pregnancy BMI												
<18.5	2.26	0	Ref.	6.15	0	Ref.	90.7	0	Ref.	103.0	0	Ref.
18.5–23.9	2.27	−0.07	0.073	6.22	−0.06	0.546	96.0	−0.3	0.901	105.9	0.1	0.970
≥24.0	2.12	−0.21	<0.001	5.97	−0.20	0.143	95.3	1.6	0.612	103.2	−1.7	0.523

β, regression coefficient; Ref, reference category.

## 4. Discussion

In this large cross-sectional study conducted in three typical urban areas of China, DHA concentrations measured by relative weight percent to total fatty acids in pregnant and lactating women were higher in coastland/lakeland women than in inland women as well as higher in mid-pregnancy than in late-pregnancy/lactation. Moreover, we observed a moderate to high degree of correlation between plasma and erythrocyte DHA.

Consistent with a previous study [8], we found DHA concentrations, whether in plasma or erythrocyte, for each participant group, were significantly higher in coastland/lakeland than in inland women, likely reflecting differences in consuming aquatic products. Relevant data of Chinese pregnant or lactating women are sparse. One small study [14] conducted in 138 late-pregnant Chinese women reported similar results for plasma choline phosphoglyceride DHA: highest in coastland, followed by lakeland, and lowest in inland. In addition, consistent with longitudinal studies [10,15], we observed that both plasma and erythrocyte DHA relative concentrations, despite the region, were significantly higher in mid-pregnancy than in late-pregnancy, probably in response to an increasing fetal needs for DHA and an increasing blood volume during pregnancy [16,17]. Meanwhile, we noticed that DHA absolute concentrations were higher in late-pregnancy than mid-pregnancy, which was probably due to an increasing synthesis during pregnancy [17]. Additionally, consistent with the findings from Stark *et al.* [18], DHA concentrations in plasma and erythrocytes increased with maternal age and education, possibly reflecting the difference in consuming aquatic products. We further compared DHA concentrations in total lipids (reported as wt. % of total fatty acids) with other populations worldwide, and found that the DHA concentrations of Chinese pregnant and lactating women were at relatively high level. Specifically, the concentrations of our inland women were higher than those of women residing in inland areas of India [19] or Germany [20], and close to or even higher than those of women residing in coastal areas of some nations like USA, United Kingdom, Denmark, Norway, Japan, or Canada [17,21,22,23,24,25]; however, the concentrations of our coastland women were slightly lower than those of Spanish (mid-pregnancy: 3.19% *versus* 3.70% in plasma) [26] and Cubans (lactation: 2.24% *versus* 2.56% in plasma and 6.07% *versus* 6.80% in erythrocyte) [27]. Besides differences in consuming aquatic products or DHA enriched supplements, the potential explanations for ethnic differences also involve DHA synthesis and metabolism, for *FADS* genotypes influence maternal DHA concentrations [28,29].

As expected, we observed a moderate to high level of positive correlation between plasma and erythrocyte DHA (Pearson’s *r* = 0.63). The correlation was even stronger (*r* = 0.73) in women with erythrocyte DHA ≥3%, but not at all in those with erythrocyte DHA <3% (*n* = 36; *r* = −0.13, *p* = 0.46); interestingly, the plasma concentrations in the two subgroups of women (2.25% *versus* 2.04%) did not differ materially. One explanation regarding the inflection point in the correlation between plasma and erythrocyte DHA was that erythrocyte could serve as a reservoir and its DHA could be transported into plasma for body need in case of a lower DHA status [30]. Another explanation was that the storage of the blood samples at −20 °C might have compromised erythrocyte DHA, especially in those with lower DHA concentrations, whereas the storage probably had no impact on plasma DHA, which in turn resulted in a flawed deviation from the linear correlation [11,12]. Therefore, the correlation identified in our study should be interpreted with caution, which remains to be confirmed in further studies.

Our study has multiple strengths. We selected three typical regions with plausible differences in DHA intake due to differences in the availability of aquatic products, and recruited three groups of women to simultaneously assess DHA status in mid-pregnant, late-pregnant and lactating women by region. Procedures in data collection and sample analyses were intensively monitored. The region- and participant group-specified sample size (~135) was the largest compared to previous similar studies (~50). However, our study also has several limitations. Firstly, the study was not longitudinal, and was conducted only in urban areas of China, possibly confining the generalization of findings. Secondly, the mean erythrocyte DHA concentrations might be slightly lower than the true values due to the temporary storage of blood samples under −20 °C [11,12]. Thirdly, the regional differences in DHA concentrations for pregnant and lactating women could not be simply generalized to the non-pregnant because the increased DHA synthesis during pregnancy was likely more pronounced in individuals with lower DHA intakes [17]. Additionally, we only focused on DHA in this study as a preliminary step to understand maternal status of polyunsaturated fatty acids (PUFAs) in our population. Further studies regarding other PUFAs are encouraged, which are critical to the understanding of the entire profile of PUFAs as well as its relationship with dietary fatty acids.

In summary, DHA concentrations of Chinese pregnant and lactating women are higher in coastland and lakeland regions than in inland areas. DHA status in our population appears to be stronger than populations from other countries as reported in the literature. DHA concentrations varied by region and participant groups, which is likely due to differences in consumption of aquatic products or changes in physiological needs for DHA.

## Supplementary Materials

Supplementary materials can be accessed at: http://www.mdpi.com/2072-6643/7/10/5428/s1.
