# Supplementary material for: DHA in Pregnant and Lactating Women from Coastland, Lakeland, and Inland Areas of China: Results of a DHA Evaluation in Women (DEW) Study
Source: Nutrients. 2015 Oct 21;7(10):8723–32. doi: 10.3390/nu7105428 (PMC4632448; doi:10.3390/nu7105428)
Supplement: Supplementary file 1 [file nutrients-07-05428-s001.docx]

**Supplementary Material**

**Table S1.** Pearson correlation coefficients (*r*) between plasma and erythrocyte relative DHA concentrations by region and participant group *.

| **Participants Group** | **All Participants** | | **Participants with** **Erythrocyte DHA ≥3%** | | **Change (%) in *r* ^†^** |
| --- | --- | --- | --- | --- | --- |
|  | ***n*** | ***r_0_*** | ***n*** | ***r_1_*** |  |
| Mid-pregnancy |  |  |  |  |  |
| Coastland | 136 | 0.495 | 133 | 0.695 | 40.4 |
| Lakeland | 133 | 0.661 | 133 | 0.661 | - |
| Inland | 138 | 0.530 | 136 | 0.550 | 3.8 |
| Late pregnancy |  |  |  |  |  |
| Coastland | 127 | 0.357 | 120 | 0.562 | 57.4 |
| Lakeland | 134 | 0.585 | 132 | 0.584 | -0.2 |
| Inland | 136 | 0.369 | 126 | 0.605 | 64.0 |
| Lactating |  |  |  |  |  |
| Coastland | 136 | 0.494 | 128 | 0.661 | 33.8 |
| Lakeland | 135 | 0.688 | 135 | 0.688 | - |
| Inland | 136 | 0.478 | 132 | 0.564 | 18.0 |

* All Pearson correlation coefficients were significant (*p* < 0.001); ^†^ Change (%) = (*r*_1_ *− r*_0_)/*r*_0_ × 100%.

© 2015 by the authors; licensee MDPI, Basel, Switzerland. This article is an open access article distributed under the terms and conditions of the Creative Commons by Attribution (CC-BY) license (http://creativecommons.org/licenses/by/4.0/).
